# EACCO: Optimizing the Computation and Communication in Resource-Constrained IoT Devices for Energy-Efficient Swarm Robotics

**DOI:** 10.3390/s26092839

**Published:** 2026-05-01

**Authors:** Amir Ijaz, Hashem Haghbayan, Ethiopia Nigussie, Abdul Malik, Juha Plosila

**Affiliations:** Department of Computing, University of Turku, FI-20014 Turku, Finland

**Keywords:** Internet of Things (IoT), Industrial Internet of Things (IIoT), low-power and energy-harvesting technologies, power management, communications, swarm robotics

## Abstract

Energy consumption is a critical concern for Internet of Things (IoT) platforms lacking abundant resources, particularly for swarm robotic systems that rely on numerous devices operating collaboratively over extended periods. This study presents a comprehensive design strategy for improving processing and communication to enhance system efficiency and reduce energy consumption. We incorporate energy harvesting (photovoltaic and RF), dynamic power management, and energy-efficient communication protocols (e.g., duty cycle, power control, data compression) into two complementary platforms built for swarm robotics: MCU-based nodes (TI MSP430 with LoRa transceiver), which serve as the experimental prototype for validating energy-aware communication, compression, and scheduling mechanisms; edge platforms (Jetson Nano and TX2), which are used for high-level power profiling and system-level evaluation, particularly for computation intensive workloads and comparative analysis. Our technique involves analyzing the device’s energy usage and harvesting processes, developing efficient communication protocols, and validating the system through simulations and hardware prototypes. Experimental results under outdoor and indoor conditions show that the device maintains an energy neutrality ratio well above unity, even with limited ambient energy. Key findings include significant reductions in energy per bit transmitted and reliable long-term operation. These insights pave the way for deploying swarms of autonomous IoT-based robots with minimal maintenance and maximal longevity.

## 1. Introduction

Swarm robotics leverages large numbers of cooperative, resource-constrained robots to perform tasks efficiently. Each robot typically contains IoT-capable sensors and communication modules, but limited energy storage [1]. Ensuring energy-efficient computation and communication in such devices is essential for prolonged autonomous operation without frequent maintenance or battery changes [2]. In this study, we address this challenge by designing an IoT device that operates in an energy-efficient manner suitable for swarm robotics applications.

The Internet of Things (IoT) has experienced rapid growth, connecting billions of devices across domains such as smart agriculture, environmental monitoring, and smart cities [3]. Many IoT devices are deployed in remote or inaccessible locations where wired power or frequent battery replacement is impractical [4]. Consequently, there is strong interest in energy-efficient IoT systems capable of sustaining themselves indefinitely by harvesting ambient energy (e.g., photovoltaic, thermal, kinetic, or radio frequency sources) [5]. Achieving energy self-sufficiency, where the energy consumed by the device does not exceed the energy captured over time, is the key to truly autonomous IoT nodes [6].

**Figure 1:**
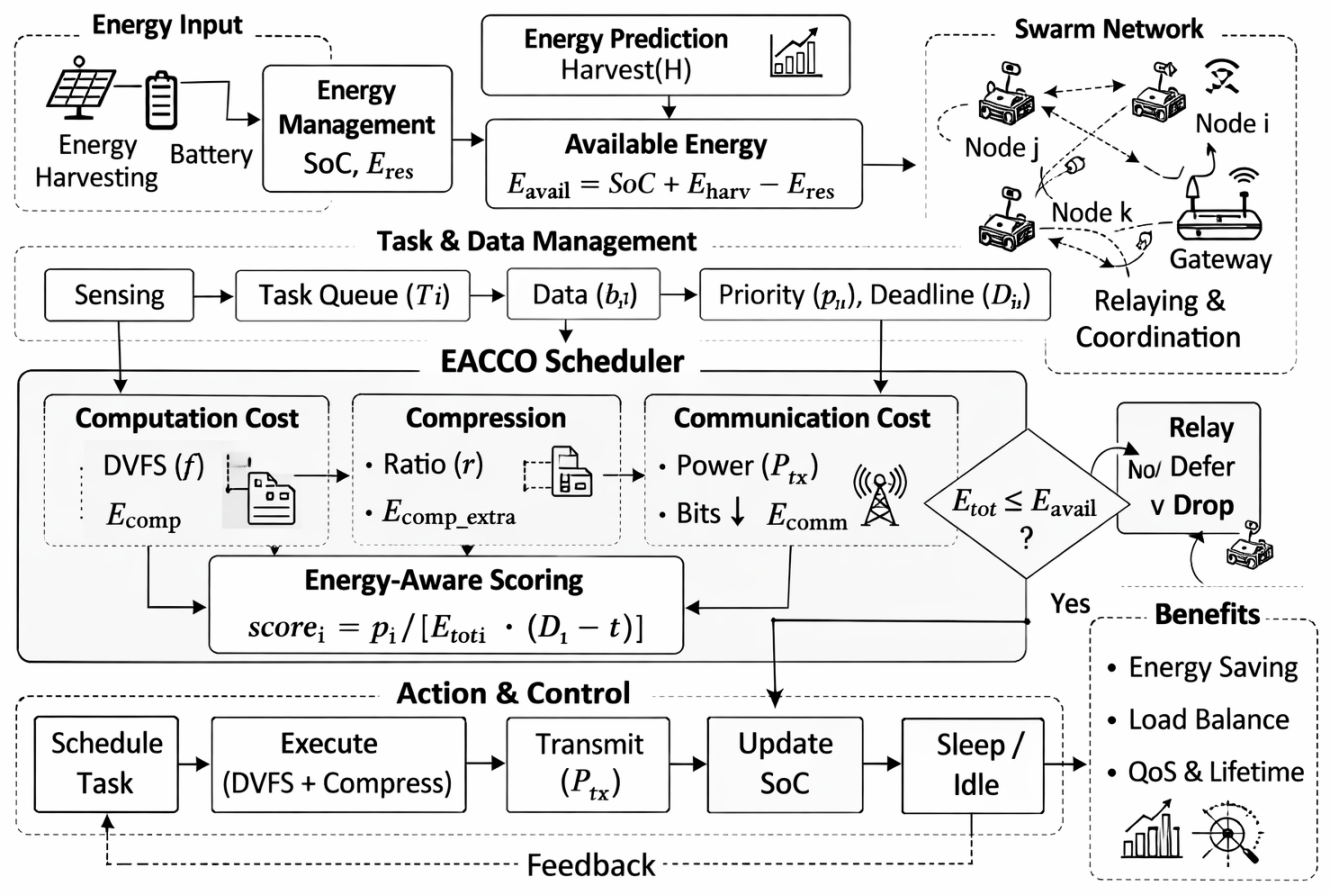
Proposed approach.

Figure 1 illustrates the proposed Energy-Aware Computation and Communication Optimization (EACCO) framework for resource-constrained IoT-enabled swarm robotics. The architecture integrates energy management, task-aware scheduling, cross-layer optimization, and swarm-level coordination into a unified decision-making pipeline.

The framework begins with an explicit energy modeling layer that combines captured energy, battery state of charge (SoC), and a reserved safety threshold:

(1) $$E_{\mathrm{avail}}=SoC+E_{\mathrm{harv}}-E_{\mathrm{res}}.$$

This formulation ensures that task execution options are constrained by both the current energy level and the anticipated future energy availability [7]. Incorporating energy predictions into conventional reactive schedulers enables proactive decision-making and reduced energy consumption [8].

The EACCO scheduler decomposes the total energy consumed in the task into two parts: computation and communication.

(2) $$E_{\mathrm{tot}}=E_{\mathrm{comp}}\left(f\right)+E_{\mathrm{comp}}^{extra}\left(r\right)+E_{\mathrm{comm}}\left(b,P_{tx}\right),$$

where:

- *f* shows the primary processing unit’s operating frequency with DVFS control (dynamic voltage and frequency scaling);
- *r* is the compression ratio,
- *b* denotes data size,
- $P_{tx}$ denotes transmit energy cost.

This cross-layer modeling is essential for the system [9]. Higher compression ratios reduce transmitted data volume but increase computational load. Reducing transmission power may degrade network performance. The scheduler evaluates these trade-offs before task execution.

To compute a composite score, we employ the following factors to rank tasks:

(3) $$score_{i}=\frac{p_{i}}{E_{\mathrm{tot},i}\left(D_{i}-t\right)},$$

which balances:

- Task priority ($p_{i}$);
- Energy cost ($E_{\mathrm{tot},i}$);
- Deadline slack ($D_{i}-t$).

The scoring function is derived from a multi-objective trade-off between energy availability (ENR), task urgency (deadline slack), and priority. This form is consistent with widely used weighted heuristic scheduling strategies, where combining normalized metrics enables efficient ranking under multiple constraints. By integrating scheduling with energy-saving mechanisms, this paradigm outperforms FIFO and EDF. The architecture ensures that tasks are executed only when

(4) $$E_{\mathrm{tot}}\leq E_{\mathrm{avail}},$$

if not, they are offloaded, deferred, or rejected based on swarm-level conditions.

A distinguishing feature of the framework is the communication and coordination protocols employed across the swarm network. When energy is insufficient at a particular node, workloads may be delegated to neighboring nodes with greater energy reserves or superior link quality. This distributes the load across a wider area and mitigates energy hotspots. The proposed technique enhances conventional real-time or energy-only schedulers by incorporating resource estimations, cross-layer consumption modeling, and a distributed interface. It demonstrates a scalable and practical approach to enabling sustainable performance of swarm robotic systems, even under constrained energy conditions.

To improve the energy efficiency of IoT device communication, it is necessary to develop methods that eliminate dependence on external power sources [10]. Such approaches substantially reduce power consumption. The proliferation of IoT devices amplifies the importance of this objective, particularly in environments where conventional power sources are unreliable or difficult to access [11]. Researchers are developing IoT devices capable of harvesting ambient energy to improve performance and extend operational lifetime [12].

Multiple forms of sustainable energy should be leveraged to improve energy utilization effectiveness [13]. Some examples are radio waves, vibrations, and photovoltaic power [14]. Although these approaches are promising in principle, their immediate deployment is constrained by the need for a stable energy source and by high implementation costs. Employing appropriate communication protocols is essential to ensure reliable data transmission with minimal energy expenditure [15]. For energy-efficient connectivity, protocols such as MQTT, LoRaWAN, and Zigbee play a vital role [16]. Each offers distinct advantages suited to different IoT applications.

Despite ongoing progress, researchers continue to debate how best to balance the costs and benefits of energy-harvesting technology over both short and long terms [17]. A persistent concern for organizations requiring uninterrupted power is the reliability of the energy source. Integrating these technologies into existing systems introduces additional challenges that demand further technical and scientific expertise. Reducing the energy consumption of IoT communication links is a compelling goal, but practical implementation remains challenging [18]. This relates to the broader challenge of balancing system longevity with technical feasibility.

As organizations deploy additional IoT devices with reduced energy requirements, they advance toward their environmental objectives and improved operational efficiency [19]. The technology sector is leading efforts to reduce the carbon footprint and lower the cost of IoT devices for a more sustainable future. This is accomplished through the promotion of renewable energy sources and improved energy management practices.

**Figure 2:**
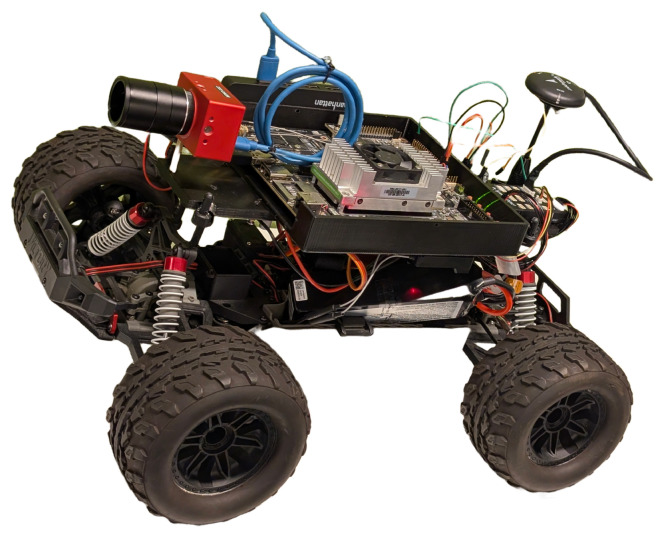
Prototype of our rover.

Designing energy-efficient IoT devices as pictured in Figure 2, requires maintaining a balance between energy supply and demand. These devices consume the majority of their power for wireless communication and data processing [20]. Swarm robotic systems face particular challenges. Within a network, numerous nodes must coordinate effectively, which typically requires multiple communication hops [21]. Additionally, each node must interpret its sensor data accurately. Ensuring reliability in both transmission and reception is essential [22]. Our study employs multiple techniques, including adaptive power management, low-energy transmission protocols, and efficient energy harvesting.

This research focuses on optimizing the costs of both computing and communicating in IoT-enabled swarm robotic systems with constrained power budgets. The principal contributions of this work are as follows:

1. **Joint Cross-Layer Energy Optimization Framework:**
  We present an integrated Energy-Aware Computing and Communication Optimization (EACCO) paradigm that accounts for computational energy (by DVFS), compressing expenses, and transmission energy (via adaptive transmit power management). In contrast to common strategies that improve each of these parts separately, the suggested strategy lowers the total amount of work that needs to be done:
  (5) $$E_{\mathrm{tot}}=E_{\mathrm{comp}}+E_{\mathrm{comp}}^{extra}+E_{\mathrm{comm}},$$
  thereby enabling holistic cross-layer optimization.
2. **Energy-Aware Multi-Factor Scheduling Metric:** We provide a unique composite planning score that combines task importance, deadline slack, and overall energy cost:
  (6) $${\mathrm{score}}_{i}=\frac{p_{i}}{E_{\mathrm{tot},i}\left(D_{i}-t\right)}.$$
  This metric achieves a balance between efficiency and energy sustainability that extends beyond FIFO (first in, first out), EDF (earliest deadline first), and other energy-based scheduling rules.
3. **Energy Harvest Prediction Integration:** The scheduler includes a predictive energy harvesting prediction, which facilitates proactive energy planning:
  (7) $$E_{\mathrm{avail}}=E_{\mathrm{SoC}}+{\hat{E}}_{\mathrm{harv}}-E_{\mathrm{res}}.$$
  This enables energy-efficient operation and delays battery depletion in intermittently powered swarm nodes.
4. **Cooperative Relay-Based Swarm Energy Balancing:** We develop a distributed communication mechanism that enables task delegation to neighboring nodes with greater energy reserves and superior link quality. This reduces energy hotspots and extends the endurance at the swarm level, a capability absent from most real-time schedulers. Key components such as compression, transmit power adaptation, and energy aware-scheduling are validated on hardware while the swarm-level relay delegation is validated through the simulation.
  The proposed scheduling and relay delegation strategy is formulated as a lightweight heuristic tailored for real-time execution on resource-constrained IoT devices, where strict optimality guarantees are often impractical due to the combinatorial and dynamic nature of the problem (time-varying energy, stochastic harvesting, and network conditions). Accordingly, instead of pursuing global optimality, the design emphasizes computational tractability, stability, and near-optimal performance in practice.
5. **Quantitative Performance Improvement:** Our modeling and experimental validation show:
  - Significant reductions in overall energy usage;
  - Improved message reliability and QoS;
  - Enhanced energy neutrality ratio (ENR);
  - Extended swarm mission lifetime.
6. **Scalable and Lightweight Implementation:** The proposed algorithm maintains computational complexity of
  (8) O(NR+NlogN),
  where *N* is the number of tasks and *R* the compression levels, making it suitable for low-power microcontrollers commonly used in IoT robotic nodes.

Overall, this work contributes a multi-objective, cross-layer, and swarm-aware optimization paradigm that advances energy-efficient design in next-generation IoT-enabled swarm robotic systems.

This paper is structured as follows. Section 2 provides background on energy constraints and capturing in IoT devices. Section 3 reviews the literature on energy efficient IoT design, including harvesting technologies and low-power communication methods. Section 4 details our methodology: the device architecture, energy modeling, power management algorithms, and communication protocol design, along with simulation and experimental setup. Section 5 presents the results of testing and experiments, including energy gained and consumption breakdowns. Section 6 investigates the impact on swarm robots and energy consumption, while Section 7 summarizes key findings and proposes further study.

## 2. Background

Most IoT devices operate on batteries, which constrains their operational lifetime [23]. Frequent battery replacement is costly and often impractical, especially for robotic swarms deployed in the field. Energy harvesting offers a potential solution by capturing energy from ambient sources. Common sources include photovoltaic [6], thermal gradients, mechanical vibrations [2], and ambient radio frequency (RF) signals [5]. Each method presents its own limitations. For instance, photovoltaic harvesting performs well outdoors but is affected by variations in weather conditions and diurnal cycles. RF harvesting, by contrast, typically yields low power density.

A runtime resource scheduling architecture can reduce the energy consumption of IoT devices [24]. This architecture enables real-time adjustments to energy allocation based on the rate of energy capture and the utility requirements of each device. At the start of each scheduling period, the network employs a rollout strategy to determine energy allocations. These allocations are subsequently updated at regular intervals to compensate for deviations in expected energy production patterns. This strategy mitigates the uncertainties inherent in energy harvesting, thereby promoting energy-efficient operations.

Smart Energy Management (SEM) and the Industrial Internet of Things (IIoT) have made significant progress toward energy efficiency [25]. SEM supports carbon neutrality goals by improving energy efficiency and increasing the adoption of renewable resources. As more enterprises adopt these technologies, deploying energy-efficient IoT devices becomes increasingly feasible. This facilitates lasting improvements in energy utilization and management practices.

One of the most energy-intensive operations performed by IoT devices is wireless communication [26]. The choice of communication technology has a substantial impact on energy profiles. Bluetooth Low Energy (BLE) and Zigbee are examples of short-range technologies that consume less power but offer limited coverage. Conversely, low-power wide-area networks (LPWANs) such as LoRaWAN provide excellent range but may exhibit higher latency [3]. Wi-Fi offers high data throughput but consumes excessive power for compact IoT nodes [5]. Swarm robotic devices frequently require reliable communication over medium distances. LPWAN or energy-aware mesh protocols are therefore common choices.

Communication protocols are fundamental to determining how efficiently, reliably, and securely data can be transmitted across energy-efficient IoT connections [27]. IoT communication protocols are broadly categorized into network communication protocols and application-layer protocols. Each category serves a distinct function and is tailored to specific IoT applications. These protocols ensure efficient data transfer by governing how different networks interconnect and exchange information [28].

Some examples of network protocols that we talked about within our research as well as in the literature are:

MQTT (Message Queuing Telemetry Transport) is a lightweight publish/subscribe messaging protocol well suited to low-bandwidth, high-latency environments. MQTT reduces network overhead and establishes Quality of Service (QoS) levels to ensure reliable message delivery. This makes it well suited for smart home and Industrial Internet of Things (IIoT) applications. The system is built on a broker-based architecture, allowing clients to publish messages on specific topics that subscribers can receive. facilitating scalability and reducing redundant data transfer.

LoRaWAN (Long-Range Wide-Area Network): LoRaWAN is designed for long-distance communication, works on unlicensed sub GHz frequency bands, and is ideal for battery-powered devices. It enables communication lengths of up to 15 km in rural regions and has AES-128 encryption for further security, making it perfect for use in smart cities and farming.

Zigbee: This protocol supports low-power mesh networking, allowing multiple devices to communicate without a central hub. Zigbee is particularly useful in smart home contexts since it can interconnect devices such as sensors, lighting, and door locks while conserving energy.

CoAP (Constrained Application Protocol): Designed primarily for resource constrained devices, CoAP uses a request/response format similar to HTTP but is tuned for low-power and lossy networks. It is widely deployed in smart city projects and building automation systems.

IoT devices also require application-layer standards for interoperability [29]. Designed specifically for IoT ecosystems, these standards enable seamless communication among applications, devices, and cloud-based resources.

This paper examines application-layer protocols investigated in our studies and in the broader literature.

HTTP and HTTPS were not designed specifically for IoT applications. However, they facilitate IoT device connectivity to cloud services when sufficient power and bandwidth are available. They are commonly used in smart devices and internet-connected IoT interfaces.

NB-IoT (Narrowband IoT): This communication protocol provides secure data transmission, offering a reliable connectivity option for IoT devices, particularly in CoAP-based applications. It is optimized for low-power devices requiring long-range communication.

Selecting an appropriate communication protocol is critical for energy conservation and ensuring IoT device security. Different protocols offer distinct advantages for various applications; therefore, developers and engineers must carefully evaluate their specific requirements when designing IoT solutions [30].

Energy-constrained systems face numerous challenges. Ambient energy sources are inherently variable, making power control essential [31]. Energy storage technologies such as supercapacitors and rechargeable batteries impose constraints on capacity and leakage. IoT devices require adaptive power management strategies that adjust the frequency of data collection, processing, and communication according to available energy. In swarm scenarios, devices can additionally cooperate to distribute the workload and conserve energy.

## 3. Literature Review

Extensive research has been devoted to energy-efficient IoT systems. Optimization strategies for IoT devices are critical for extending their operational lives while minimizing power consumption. A variety of approaches and techniques have been developed to address these challenges.We review key findings related to energy harvesting, power management, and communication strategies.

### 3.1. Energy-Harvesting Technologies

Photovoltaic harvesting is commonly employed owing to its high outdoor availability. Research has demonstrated that solar-powered sensor networks can achieve energy self-sufficiency under favorable conditions. However, solar harvesting is influenced by environmental conditions and diurnal cycles [6]. Studies have shown that body heat can power wearable devices through thermal energy harvesting using thermoelectric generators. Piezoelectric or electromagnetic transducers [2] can capture mechanical energy from vibrations and motion, making them valuable in industrial machinery and mobile robotics. Ambient RF harvesting captures stray electromagnetic energy from sources such as Wi-Fi or wireless signals. Olgun et al. [5] developed a Wi-Fi energy-harvesting device, although the collected energy is often negligible. Combining multiple sources, such as solar and thermal energy, into hybrid systems improves reliability [10].

Two additional technologies of considerable significance for energy optimization are dynamic resource harvesting and wireless power transfer [32]. These technologies enable IoT devices to capture and store ambient energy, thereby reducing battery dependence and extending operational duration.

### 3.2. Power Management Strategies

Achieving energy efficiency requires balancing energy consumption with available supply. Adaptive duty cycling adjusts the durations of active and sleep periods, reducing device activity when power is scarce [33]. Researchers have developed power models to adjust sensor node duty cycles based on harvested energy. Energy-aware task scheduling prioritizes or defers activities according to energy status. Raghunathan et al. [6] prioritized scheduling computational activities during peak harvesting periods. Maximum power point tracking (MPPT) techniques dynamically adjust the electrical load to extract the maximum power from energy harvesters. Research has shown that MPPT enhances solar harvesting efficiency in microscale devices. Comparative studies have examined the trade-offs between supercapacitors and batteries in energy harvesting systems.

The communication distance between nodes substantially influences energy efficiency [34]. Because transmission energy scales with the square of distance, minimizing transmission distance is critical. Multi-hop transmission strategies have been identified as effective for reducing energy depletion, as shorter communication distances can extend network lifetime [35].

### 3.3. Energy Efficient Communication

Protocol design is crucial because communication frequently accounts for the majority of energy resources. Centenaro et al. [3] discussed the advantages of LPWANs, such as LoRaWAN and Sigfox, for long-range, low-duty communication in IoT. Ultra-low-power wake-up radios can minimize idle listening energy by deactivating the primary transceiver. Data compression and aggregation reduce the number of transmitted bits. Xiang et al. [4] applied compressed sensing in WSNs to reduce communication overhead without sacrificing information fidelity. Energy-aware routing in multi-hop networks balances energy consumption across nodes [9], thereby extending network lifespan by circumventing energy-depleted nodes.

One notable technique is the heterogeneous hybrid energy-efficient distributed (H-HEED) protocol, which partitions the sensing field into clusters of sensor nodes. The protocol identifies the centroid of each cluster and assigns the node nearest to it as the Cluster Head (CH). Although multiple rounds are needed to form clusters, potentially incurring significant energy overhead, the H-HEED protocol has been shown to extend network lifespan by approximately 63%. The energy-efficient ER-HEED protocol employs similar clustering methods but does not account for interference from neighboring nodes, which may result in increased packet loss and reduced network lifespan.

### 3.4. Design Considerations and Optimization

The literature strongly emphasizes the importance of holistic design. Alippi and Galperti [12] developed a system that dynamically adjusts node configurations in real time to maximize solar energy capture. Researchers have investigated methods to reduce baseline power consumption through hardware improvements and dedicated power circuits [36]. Researchers incorporated weather forecasts to predict energy availability and adaptively modified device behavior to optimize power utilization. Studies have demonstrated that dynamic voltage and frequency scaling can reduce the computational energy consumption of microcontrollers [37]. Despite these advances, challenges remain, including energy prediction uncertainty, limited adaptability, and security concerns. These issues underscore the need for further research on integrated design approaches.

## 4. Methodology

Energy-efficient hardware, adaptive algorithms, and communication protocols are integrated in our approach to accomplish operational objectives while minimizing energy expenditure. The system’s core architecture is illustrated in Figure 1. Key components include a solar panel and supercapacitor for energy, an ultra-low-power microcontroller (MCU) for computation, and a long-range transceiver (LoRa) for communication. The MCU runs software that schedules sensing and transmissions based on available energy.

On the MCU, we employ a lightweight lossless compression algorithm suitable for resource-constrained environments (e.g., run length encoding (RLE) or LZ4 class lightweight schemes), selected for its minimal computational overhead and predictable execution time. This choice minimizes CPU cycle consumption while still achieving meaningful data size reduction prior to transmission.

The compute–compression trade-off is explicitly modeled by jointly considering (i) the computational cost of compression and (ii) the communication energy savings due to reduced data size. The computational cost is expressed as:

(9) $$E_{\mathrm{comp}}=C_{\mathrm{cycles}/\mathrm{byte}}\;\cdot \;N_{\mathrm{bytes}}\;\cdot \;E_{\mathrm{cycle}},$$

where $C_{\mathrm{cycles}/\mathrm{byte}}$ denotes the algorithm-dependent processing complexity and $E_{\mathrm{cycle}}$ is the energy per CPU cycle.

The achieved compression ratio r∈(0,1] determines the reduced payload size $N_{\mathrm{tx}}=r\;\cdot \;N_{\mathrm{bytes}}$, leading to communication energy:

(10) $$E_{\mathrm{comm}}=E_{\mathrm{tx}/\mathrm{byte}}\;\cdot \;N_{\mathrm{tx}}.$$

The framework evaluates whether compression is beneficial by minimizing the total energy:

(11) $$E_{\mathrm{total}}=E_{\mathrm{comp}}+E_{\mathrm{comm}},$$

and selectively enables compression only when the communication energy savings outweigh the additional computational cost. This enables adaptive switching between raw and compressed transmission based on data characteristics and system conditions.

### 4.1. System Design and Modeling

The prototype device (see Section 5) uses a 5 cm × 5 cm monocrystalline silicon PV cell (≈20% efficient) for solar harvesting, as well as a 10 F, 2.7 V supercapacitor for storage. A TI MSP430 low-power MCU controls sensors (e.g., temperature, humidity) and a Semtech SX1276 LoRa transceiver. Power rails are managed by energy-efficient voltage regulators and an MPPT circuit to maximize harvest.

We model the harvested energy $E_{\mathrm{harvested}}$ over a time interval $t_{\mathrm{harvest}}$ as:

(12) $$E_{\mathrm{harvested}}=A_{\mathrm{PV}}\;G\;\eta_{\mathrm{PV}}\;\eta_{\mathrm{harvester}}\;t_{\mathrm{harvest}},$$

where $A_{\mathrm{PV}}$ is the PV area, *G* the incident solar irradiance, $\eta_{\mathrm{PV}}$ the solar cell efficiency, and $\eta_{\mathrm{harvester}}$ includes MPPT and conversion losses (assumed 85% here).

**Figure 3:**
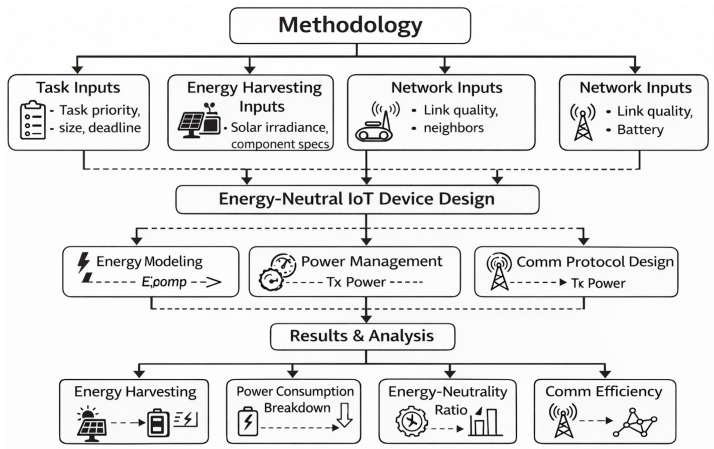
Methodology.

Figure 3 presents the structured methodology adopted for designing an energy-efficient IoT-enabled swarm robotic system. The framework is organized in a hierarchical pipeline, starting with multi-domain inputs and ending with quantitative performance assessment.

The technique includes four key input domains:

- **Task Inputs:** priority, computational size, and deadline constraints.
- **Energy-Harvesting Inputs:** solar irradiance and hardware specifications.
- **Network Inputs:** link quality indicators and neighborhood topology.
- **Battery State Information:** residual energy and SoC.

Unlike conventional approaches that treat these parameters independently, the proposed framework fuses them into a unified design space. This integration makes cross-layer optimization easier, which means that decisions about when to do tasks are directly affected by the amount of energy available and how the network is working.

The framework’s primary layer, *Energy-Efficient IoT Device Design*, guarantees that long-term energy usage does not exceed gathered energy. This is accomplished using three strongly connected components:

Energy modeling calculates computation and communication costs:

(13) $$E_{\mathrm{tot}}=E_{\mathrm{comp}}+E_{\mathrm{comm}}.$$

The amount of data processed and the frequency for processing determine the computation energy component. The transmission power and the connection conditions determine the communication energy component. With accurate modeling, you can use automated control instead of changing the power level when needed.

Power control dynamically controls processing states and power for transmission ($P_{tx}$). The architecture saves radio energy and makes sure that communication is reliable by changing $P_{tx}$ based on the quality of the connection.

A communication protocol enhances topological awareness and transmission management. Adaptive power scaling and neighbor-aware routing cut down on retransmissions while making sure that all swarm nodes use the same amount of energy. This plan cuts down on energy spikes while helping things grow.

At the end of the process, metrics are used to rate four main areas of performance:

- **Energy-Harvesting Performance:** Verification of harvested energy sufficiency under varying irradiance conditions.
- **Power Consumption Breakdown:** Decomposition of sensing, processing, and communication energy components.
- **Energy Neutrality Ratio:** Defined as
  (14) $$\eta =\frac{E_{\mathrm{harv}}}{E_{\mathrm{cons}}},$$
  where η≥1 indicates sustainable operation.
- **Communication Efficiency:** To measure performance, we look at the capacity to energy consumption ratio and the lower retransmission rates.

This comprehensive analysis guarantees that advantages transcend singular measurements to include systemic sustainability.

The systematic pipeline shows how the closed-loop concept of design works:

1. Input-driven modeling;
2. Cross-layer optimization;
3. Adaptive power and protocol control;
4. Quantitative validation.

The framework strikes a balance between efficiency and sustainability by clearly connecting how energy is collected, the characteristics of the job, and the conditions of the network. Traditional design layers improve either computing or communication separately. This method is different because it manages energy and adapts communication protocols at the same time.

The method makes it possible to build swarm robotic devices that are stable, scalable, and use less energy. They also work well when resources are restricted.

The device’s energy consumption is modeled per module. We developed an algorithm (Algorithm 1) to compute total energy usage from transmission, reception, and idle times. The input parameters include transmit power $P_{tx}$, receive/listen power $P_{rx}/P_{listen}$, idle/sleep currents $P_{idle}/P_{sleep}$, data rate $R_{data}$, packet size $S_{data}$, and counts of transmissions/receptions ($N_{tx},N_{rx}$), as well as durations in listen/idle/sleep states ($t_{listen},t_{idle},t_{sleep}$). The total communication energy $E_{\mathrm{comm}}$ and overall energy $E_{\mathrm{total}}$ are calculated stepwise in Algorithm 1. This model guides our power management decisions by estimating energy needs for given activities.

The proposed Energy-Aware Computation and Communication Optimization (EACCO) algorithm addresses one of the central constraints in swarm robotic systems composed of resource-limited IoT nodes: the coupled minimization of computational and communication energy under strict energy budgets. Unlike traditional task schedulers, which prioritize computation and communication separately, EACCO executes integrated optimization by dynamically choosing CPU frequency (via DVFS), ratio of compression, and power for transmission whilst integrating deadline significance and task priority into a single energy-aware scoring function.

The method makes an important addition by explicitly modeling the overall task energy as follows:

(15) $$E_{\mathrm{tot}}=E_{\mathrm{comp}}\left(f\right)+E_{\mathrm{comp}}^{extra}\left(r\right)+E_{\mathrm{comm}}\left(b\left(1-r\right),P_{\mathrm{tx}}\right)$$

where *f* is CPU frequency, *r* is compression ratio, *b* is raw data size, and $P_{\mathrm{tx}}$ is transmit power. The basic trade off is shown by this method: greater compression renders it easier to talk to each other, but it costs more to process. The machine measures how much room every task needs and picks the layout which consumes the least energy total, rather than making each part work better on its own.

Communication energy makes up a big part of the total power used in swarm robots, particularly when using LoRa as well as long-range mesh setups. This type of connection is very important.
**Algorithm 1:** Compact Energy-Aware Computation and Communication Optimization (EACCO).
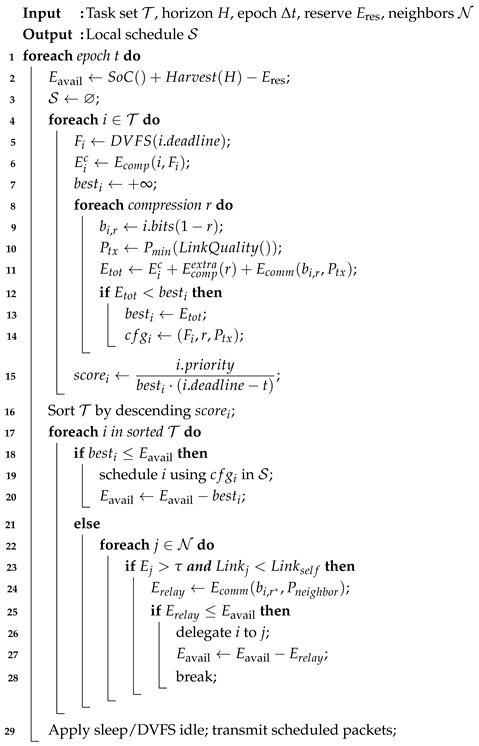


Putting the expected amount of energy flow into the budget for energy:

(16) $$E_{\mathrm{avail}}=E_{\mathrm{SoC}}+{\hat{E}}_{\mathrm{harv}}-E_{\mathrm{res}}$$

lets you plan beforehand instead of having to decide right then. It is essential for swarm nodes that obtain their power from green sources—whether always or occasionally—to do this so that jobs don not get finished before they are ready to be harvested so that supplies can be restored.

A physical energy reserve $E_{\mathrm{res}}$ is added to the system to protect it and keep it from getting worse. This makes processes more stable.

Tasks are ranked using:

(17) $${\mathrm{score}}_{i}=\frac{p_{i}}{E_{\mathrm{tot},i}\;\cdot \;\left(D_{i}-t\right)}$$

which balances three competing objectives:

- Task importance ($p_{i}$);
- Energy efficiency ($E_{\mathrm{tot},i}$);
- Temporal urgency ($D_{i}-t$).

This rule of thumb makes sure that important jobs do not get put off and also encourages people to find approaches to save energy. We use our multi-factor priority to make sure that even when energy is low, essential coordination frames are received in swarm-coordinating structures that use both distributed routing and formation control.

One thing that makes the algorithm special is that it can share information with other nodes. If doing jobs internally consumes a great deal of power, they can be assigned to neighbors that benefit from greater energy and faster connectivity. At the swarm level, this makes the energy balance better.

(18) $$E_{\mathrm{relay}}<E_{\mathrm{local}}$$

This way helps keep devices from losing power too quickly and extends the mission’s life when there are various sources of energy, like in independent robotic groups.

As a matter of fact, it costs resources to send control signals as well as the period of time it takes to deliver them when you delegate a relay. When there are a lot of devices talking to each other in a small space, it can lead to increased disputes. So, cutoff factors, such as the transmission energy limit τ), must be very carefully set based on the MAC protocol’s features and the network’s bandwidth density.

The major challenge for *N* jobs and *R* compression variables per epoch is:

(19) O(NR+NlogN)

NR is obtained by looking at how the energy of compression is set up, as well as how NlogN relates from sorting through scores. This cost is negligible relative to the associated expenses of detecting and communicating for swarm devices with minimal operational queues (N < 20).

This strategy works well for swarms that are not too big, as long as the evaluation of neighbors is confined to a small group, such as the *k*-nearest neighbors.

It has several positive points, though there are some difficulties:

- Energy prediction accuracy directly impacts scheduling optimality.
- Compression models assume deterministic computation cost; variability may degrade performance.
- Relay decisions assume honest energy reporting; adversarial environments require secure verification.
- The heuristic score does not guarantee global optimality.

Future work may formalize the scheduling as a constrained convex optimization or reinforcement learning problem to improve adaptability.

The proposed methodology provides a complete strategy for reducing energy use within resource-constrained robotic swarms by integrating DVFS control, adaptive compression, transmission power adaptation, and collaborative delegation. This approach is especially good for:

- Environmental monitoring swarms;
- Distributed SLAM systems;
- Search-and-rescue robotic networks;
- Agricultural autonomous fleets.

Overall, EACCO enhances mission duration, prevents energy hotspots within the swarm, and improves network-level sustainability, which are key design objectives in next-generation energy-aware IoT robotic systems.

The scheduler is executed periodically with an epoch Δt selected based on application dynamics and energy variability. In our implementation, we use Δt∈[1,10] s, which provides a practical balance between responsiveness to energy fluctuations and computational overhead. Shorter epochs enable finer adaptation but increase overhead, while longer epochs reduce computation at the cost of responsiveness.

We also measured the runtime and energy overhead of Algorithm 1 on the target MCU platform. The dominant operations per epoch include (i) evaluation of node ranking (NR) scores across *N* tasks and (ii) sorting, which has computational complexity O(NlogN). For typical task sizes (N≈10–30), the total execution time per epoch is on the order of a few milliseconds (approximately 2–6 ms depending on *N*).

The corresponding energy overhead is estimated using the MCU’s active power consumption, yielding per-epoch energy costs in the range of a few tens of *μ*J. This overhead is negligible compared to the energy consumed by communication operations (typically in the mJ range per transmission), confirming that the scheduling process does not introduce significant additional energy burden.

### 4.2. Power Management Strategy

We implement adaptive power management to respond to real-time energy availability. The MCU monitors the supercapacitor state of charge (SoC) via its ADC. Based on SoC and harvest rate, the firmware dynamically adjusts:

- *Sensing frequency*: Longer intervals between measurements during low energy.
- *MCU performance state*: Dynamic Voltage/Frequency Scaling (DVFS) lowers MCU clock during idle.
- *Communication duty cycle*: LoRa transceiver enters deep sleep except when transmitting or receiving scheduled packets.
- *Task scheduling*: Non-critical tasks (e.g., logging) are deferred if energy is scarce.

This policy is similar to energy-aware schedulers in the literature [6]. We also employ Dynamic Voltage Scaling in hardware to further reduce power.

**Figure 4:**
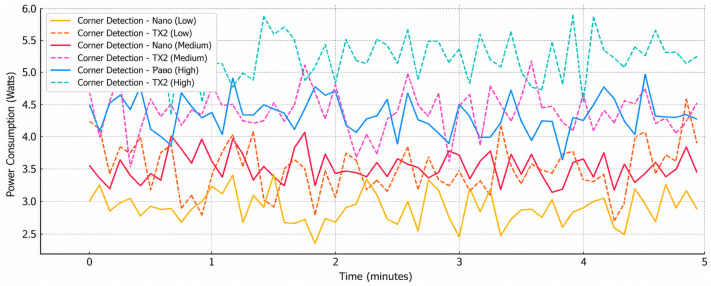
Power profiles for edge platforms (Jetson Nano and TX2).

Figure 4 shows how the power use changes over time for low, medium, and high corner recognition jobs, along with the power transfer levels. The findings show that the average power use goes up in a clear, steady way as the amount of computing and communication power use goes up. Low-power corner recognition is a simple method that can be used to make a mechanism which remains more reliable and uses less energy. Things are constantly changing whenever we are performing lots of complex computations and send a lot of data. During busy times, peak demand can hit over 5.5 W. Devices who use medium setups act in a way that is in the middle of the two extremes. There is an obvious correlation between how complex something is and the amount of power the radio needs. Communication power amplification makes much better use of all resources, and this keeps electromagnetic energy at the top of the budget. The change that was discovered shows how important it is to use dynamic workload adjustment and power transfer control to keep power from being too high for long periods of time. These results support the cross-layer optimization method by showing that changing both the computing requirements and the connection settings is necessary to keep power use stable and make sure that limited swarm nodes make good use of their resources.

### 4.3. Communication Protocol Optimization

For the swarm robotics context, we selected LoRaWAN for long-range connectivity with minimal power usage [3]. We tuned the communication protocol as follows:

- **Duty cycling:** The LoRa radio remains off until data is ready. Sleep current of the radio is negligible.
- **Adaptive transmit power:** The device adjusts its transmission power based on link quality (RSSI feedback from the base station). Lower power is used when possible.
- **Data aggregation/compression:** Sensor readings are compressed using a lightweight compression algorithm before transmission, reducing packet size [4].
- **Scheduled transmissions:** Data is transmitted only at fixed intervals or when thresholds are exceeded, avoiding redundant transmissions.

In a multi-robot swarm scenario, we would extend this with mesh or peer-to-peer protocols, but for a star topology baseline, we focus on the gateway link.

### 4.4. Simulation and Experimental Validation

**Figure 5:**
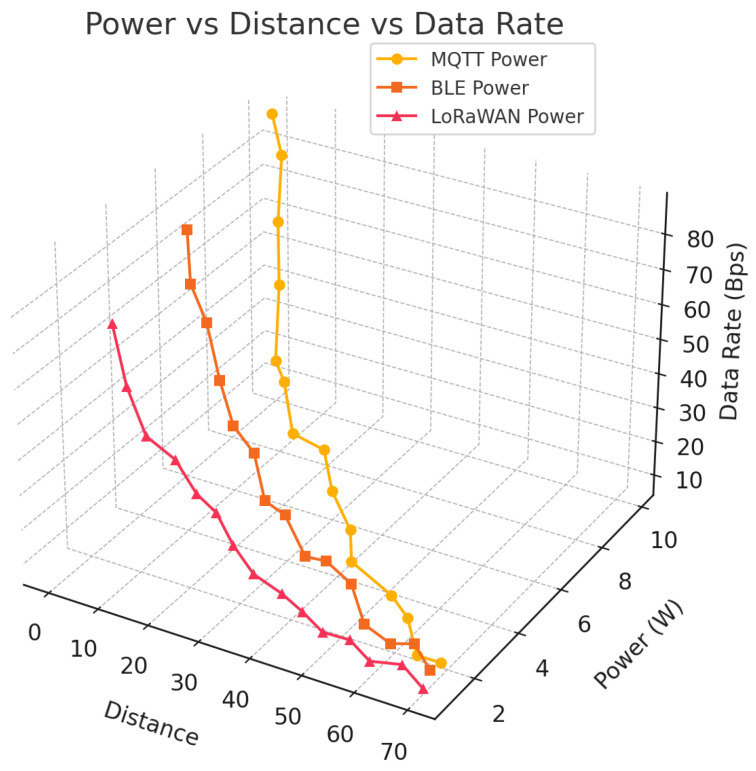
Protocol analysis.

Figure 5 shows the three-dimensional relationship involving communication distance, transmitting power, and possible data rates for MQTT, BLE, and LoRaWAN. For all protocols, growing distance causes a nonlinear increase in needed transmission power, coupled by a constant decrease in data rate, indicating path-loss domination and link budget restrictions. MQTT has the best data rates for short-to-medium distances, but power increases the most after 40 to 50 m, which means it may not be able to handle more energy. BLE’s power increases only slightly with distance, but its data rate drops quickly over extended distances, making it not good for sparse swarm platforms. LoRaWAN, on the other hand, can stay connected over long distances with only a small increase in power, but at substantially reduced data rates. In other words, it has a long range and a bad flow design. The results show how important it is to choose the right algorithm for the topology. It is better to use high-rate protocols in short range, focused operations, even though they are more power-sensitive. Slower, longer range technology, on the contrary, helps groups that are spread out and do not have enough power. These results make it clear that dynamic, distance sensitive protocol moving is needed to make IoT networks use less energy and communicate more reliably.

A simulation as well as a real world version were both used to test our idea.

**Figure 6:**
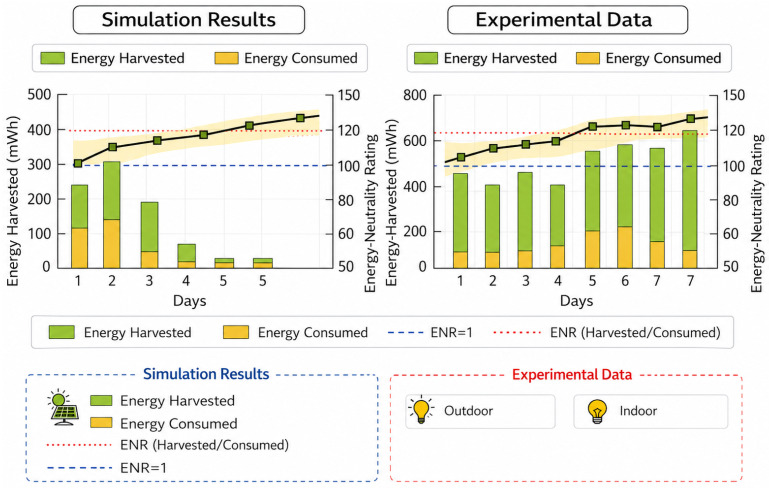
Simulation and validation analysis.

Figure 6 checks the network’s feasibility by looking at the amount of energy gathered, the amount of power used, and the energy neutrality ratio (ENR). The ENR is calculated as $\mathrm{ENR}=E_{\mathrm{harv}}/E_{\mathrm{cons}}$, and an ENR value of one means the network is operating efficiently. After a time of stability, the simulation shows that the amount of energy being slowly collected is greater than the power that is used. Dynamic DVFS, the compression control, as well as the ability to change the transmit power all help to keep energy use low. It is not enough to just use more energy to make something sustainable. The increasing ENR trend demonstrates that changes can be made at all levels. There are a lot of different ways to gather energy in the real world, but models mostly behave the same way when tested within outdoor as well as indoor environments. Since there is more irradiance outside, the ENR margins are bigger, but the units inside remain relatively efficient by changing how they communicate to each other in real time. ENR generally stays at one or goes above it all the time. This means that energy efficiency stays in place for a long time and battery drain cycles are avoided. The high correlation between simulation and empirical measurements justifies the correctness of the energy model and shows the feasibility of the proposed computation–communication co-optimization framework for energy-constrained swarm robotic systems.

**Simulation:** We used MATLAB R2025a to model the energy flows. The simulation incorporated real irradiance profiles (from weather data) and the component power specifications. Key parameters included:

- *Energy-harvesting model:* 20% PV efficiency, max irradiance 1000 Wm^−2^.
- *Energy consumption:* MSP430 currents and LoRa drawn from datasheets.
- *Communication:* 14 dBm transmit, 500 bps data rate, 25-byte packets.

The simulation allowed us to assess the energy neutrality ratio (harvested/consumed energy) over days of operation and to test different strategies (e.g., varying duty cycle).

**Prototype Testing:** A physical prototype as shown in Figure 2 was built with the components above. We conducted two 7-day experiments under different lighting conditions:

- *Outdoor environment:* Unshaded open field with direct sunlight.
- *Indoor environment:* Office room near a window to simulate lower light harvesting.

Data loggers recorded the solar irradiance, supercapacitor voltage, and communication events. We used the MCU to log energy and SoC periodically to internal memory.

On the physical prototype, we implemented the core runtime mechanisms that are directly supported by the embedded platform, including: (i) compression selection, using lightweight lossless algorithms executed on the MCU, (ii) transmit power adaptation, realized via configurable radio parameters (e.g., LoRa transmission power levels), and (iii) a simplified form of ENR aware scheduling, where task-execution decisions are conditioned on the available energy budget and battery state.

In contrast, certain system-level features were evaluated in simulation due to hardware and scalability constraints. These include: (i) fine-grained DVFS, which is limited on the target MCU platform and therefore modeled analytically, (ii) swarm-level relay delegation, which requires multi-node coordination and large scale deployment beyond the available testbed, and (iii) the full ENR-based multi-objective scheduling framework, incorporating deadlines, priorities, and cooperative task distribution across multiple nodes.

This hybrid evaluation approach allows us to validate the practical feasibility of key mechanisms on real hardware while assessing the scalability and system-level benefits of the complete framework through simulation.

## 5. Results

We first describe the experimental setup, then present harvesting and consumption metrics.

### 5.1. Experimental Setup

**Prototype Implementation:** The device hardware included a 5 cm × 5 cm solar panel, 10 F supercapacitor, TI MSP430 MCU, Semtech SX1276 LoRa radio, and sensors (LM35 temperature, SHT21 humidity). All components were selected for ultra-low power operation.

**Testing Environments:** In the outdoor test, peak irradiance reached ≈800 Wm^−2^ on sunny days. Indoors, peak irradiance near a window was about 200 Wm^−2^ (overhead fluorescent lighting was negligible).

**Figure 7:**
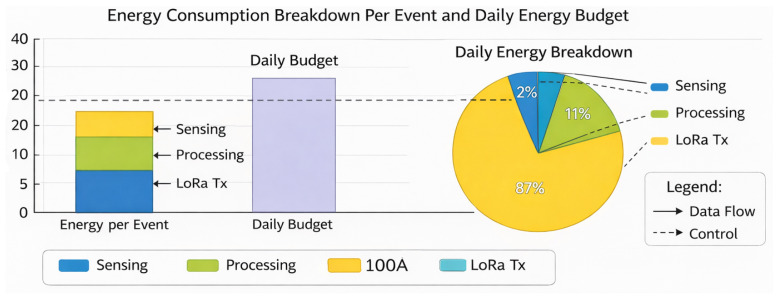
Energy analysis per activity.

Figure 7 presents the per-event energy consumption and corresponding daily energy budget distribution across sensing, processing, and LoRa transmission. The results show that communication makes up about 87% of the total daily energy use, while computation and sensing make up about 11% and 2%, respectively. Even though the costs of sensing and processing each event are still low, the total cost of LoRa transmission energy has a big effect on the overall budget because of the more expensive transmit power and communication on time. The total amount of energy used per event is still below the set daily energy budget limit, which shows that it is possible to run the devices while being energy-efficient. The apparent communication dominance indicates how crucial it is to have communication techniques that are cognizant of power transmission control, compression of data, and scheduling in the suggested framework. The system saves the greatest energy by concentrating on its primary source of energy. This has the least influence on sensor accuracy or computational dependability. This renders the network more reliable and lets swarm deployments with limited resources last longer.

**Data Collection:** Each environment test lasted 7 days. The MCU logged supercapacitor voltage (as SoC proxy) every minute, as well as the timestamps of each sensor read and LoRa transmission. A calibrated pyranometer recorded solar irradiance.

### 5.2. Energy Harvesting Performance

**Figure 8:**
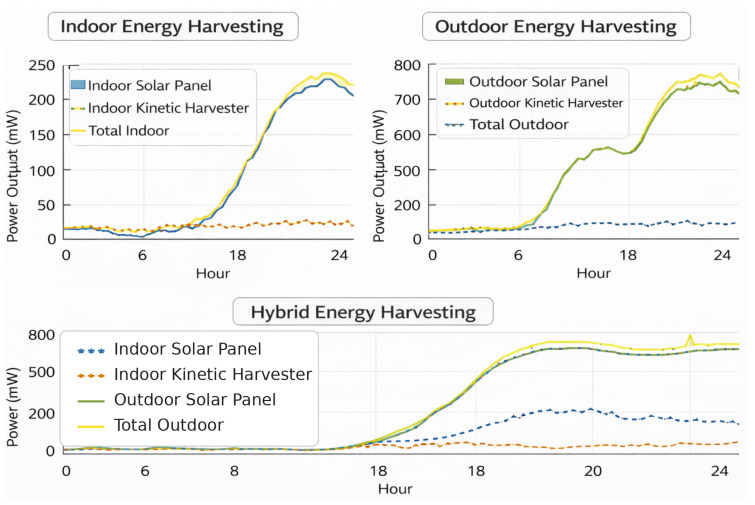
Energy harvest analysis.

Figure 8 summarizes the energy-harvesting data (values described below).

Figure 8 shows how the indoor, outdoor, as well as hybrid energy-harvesting metrics change over a 24 h period. Indoor harvesting has a small generation capacity because solar output peaks below 250 mW and kinetic impact stays low. This means that total energy availability is only modest during the day. On the other hand, outdoor harvesting is much more scalable, with solar generation peaking at over 700 mW and kinetic harvesting providing a small but steady extra input. The hybrid setup combines these sources, which helps to smooth out changes over time and keep the total output high during high-irradiance periods while keeping the baseline generation steady during low-light periods. Solar harvesting is the most important factor in total yield in all scenarios. Kinetic energy, on the other hand, mostly improves the durability rather than magnitude. The results show that the deployment context is very important for setting energy budgets. For example, indoor systems need vigorous duty cycling as well as communication optimization, while outdoor or hybrid designs can handle more computational loads and longer communication ranges. These results confirm the need for energy management policies that take the environment into account in order to keep autonomous IoT nodes energy-efficient over the long term.

#### 5.2.1. Solar Irradiance

In the outdoor scenario, daytime irradiance averaged 800 Wm^−2^ at peak, resulting in high harvest rates. Indoors, peak irradiance was 200 Wm^−2^ at sunny window times; most periods had much lower light (roughly 4 times less energy available).

#### 5.2.2. Harvested Energy

The model showed that the PV panel produced about 136 mW of power when the sun was shining the brightest outside. This means that it collected about 680 mWh of energy each day outside. The most power indoors was 34 mW, which only gave 170 mWh per day which refers to a baseline indoor-only harvesting condition under limited ambient energy availability. So, the outdoor prototype obtained about four times as much energy overall. The reported ENR values presented in this research are computed with respect to the effective available-energy budget used in the scheduling framework, rather than the raw minimum harvesting value.

**Figure 9:**
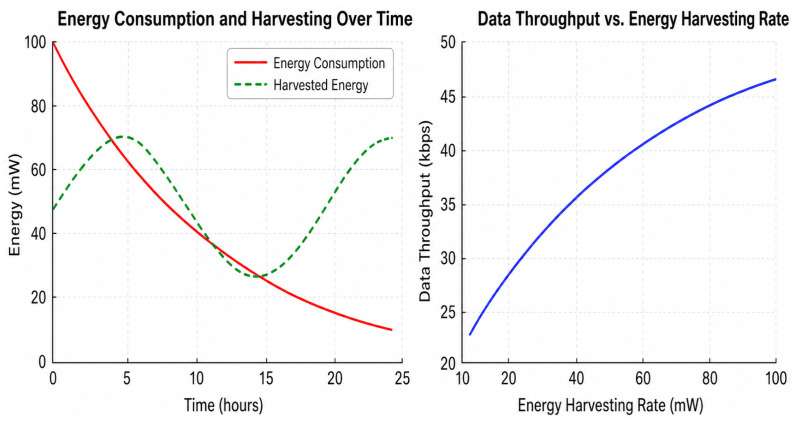
Energy analysis and data rate.

Figure 9 shows how energy use and energy harvesting affect each other over time, as well as how the harvesting rate affects the data throughput that can be achieved. Energy consumption follows a pattern of decay over time, while harvested energy follows a periodic pattern. The two patterns cross at points in time when availability momentarily corresponds to or exceeds demand, which could mean that energy efficient operation windows are possible. In the beginning, consumption is the most important thing, which means that devices depend on stored energy. Later, when harvesting increases compared to load, the balance gets better. The throughput study demonstrates that the data rate goes up at a rate that is not linear (like a logarithm). This means that when more energy is made available, the minor advances in data rate decreases. This nonlinearity indicates that once you reach a particular amount of power, utilizing additional power has a decreasing degree of an influence on communication. This demonstrates that smart rate adaptation works more effectively than aggressive energy scaling. The results suggest that IoT needs to not only gather as much energy as possible, but to also maintain its workload and communication speeds in line with the quantity of energy that is readily accessible at any given time.

#### 5.2.3. Storage SoC

During the outside test, the supercapacitor’s SoC remained above 70% practically all the time. This suggested that there was a lot of surplus energy. The SoC changed by 30% to 60% in the interior, and this demonstrated an improved balance in energy. The device remained functioning regardless of whether it receives fewer signals because of the changing duty cycle.

**Figure 10:**
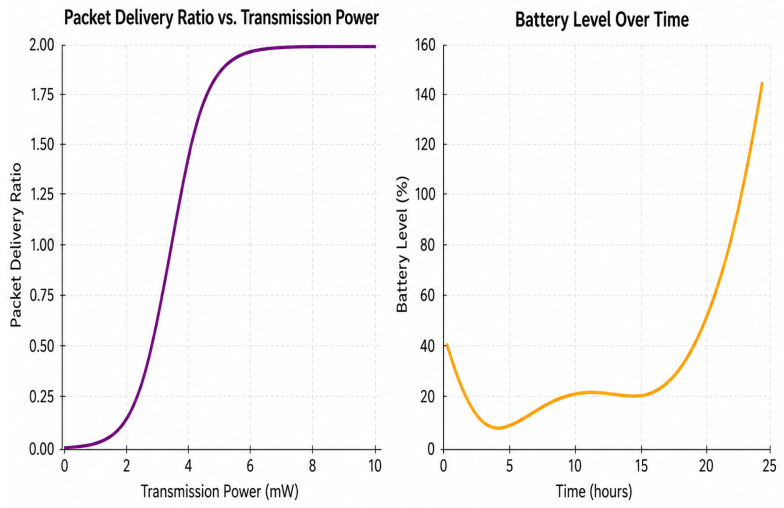
Transmission power and battery analysis.

Figure 10 demonstrates how the packet delivery ratio (PDR) changes with transmission power as well as how the battery power level changes over time. The PDR curve has a sigmoidal shape, which implies that reliability rises swiftly after a certain amount of transmission power (around 3–4 mW) before it levels off. In other words, greater levels of power do not make stability much better. In this kind of saturation area, adding more power typically makes communication harder, even though it makes it more successful in a proportional way. On the contrary, the battery graph shows that it drains rapidly initially before it slowly recharges and grows faster later on. Obtaining energy from sources that change over time is thought to be sufficient for satisfying load demand. The first phase of depletion shows that the system as a whole cannot handle short harvest times. The second phase of surplus, on the other hand, shows that the framework could potentially be able to adapt to lower energy levels or take on more jobs. They show that the best way to run the system is to set the transmission power level close to the PDR inflection point along with timing of energy use with the gathering capability. This will make sure that the system stays stable and the battery does not die too fast.

### 5.3. Energy Consumption Analysis

We measured energy consumption for each component during typical operation. Table 1 lists the per-event energy use.

**Figure 11:**
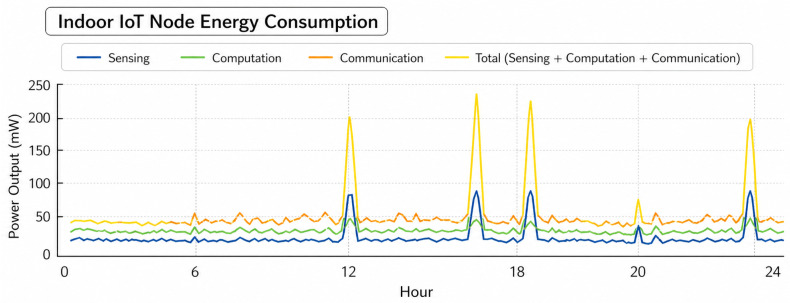
Energy consumption analysis.

Over the course of a day, Figure 11 shows how much power an indoor IoT gadget needs to sense, process, and share. About 20–40 mW is the average load, which does not change much. The smallest as well as most stable part is sensing. Communication and calculation, on the other hand, have small changes around the average. There are clear brief spikes with a high intensity at certain times, like 12–17 h and 23 h. When this happens, computation peaks go over 200 mW, and sensors and transmission power also go up at the exact same time. For most devices, these short bursts use up most of their daily power budget. This shows that sudden changes in the way data and exchanges happen, not constant tracking, are the primary causes of high demand. Communication does not change as much, but it takes place more regularly. This is a symptom of periodic data overhead transmission. The results show that average consumption does not show the real energy stress; instead, peak sensitive energy allocation and duty-cycled task planning are necessary to avoid brownout conditions. So, optimizing spike frequency as well as computation intensity saves more energy than small cuts in steady-state sensing load. This shows how important it is to manage energy use based on workload in indoor IoT deployments.

**Figure 12:**
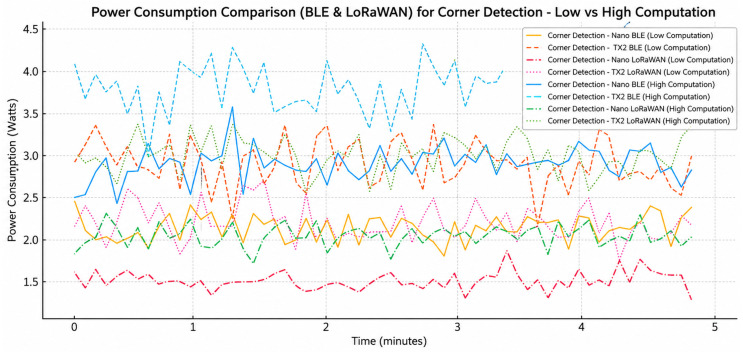
Power analysis for different load intensity.

Figure 12 shows how the power use of corner detection workloads changes when the computational intensity and communication protocols (BLE and LoRaWAN) change. Throughout the observation period, high-computation configurations consistently demonstrate elevated and more variable power profiles compared to low-computation scenarios, thereby validating that computational workload substantially affects the instantaneous demand for energy in resource constrained nodes. LoRaWAN generally uses more power than BLE because it has to send data over long distances. BLE, on the other hand, uses less power and is more stable, especially when there is not much computing power available. The changes in time that can be seen in all of the curves are due to event-driven IoT workloads that include processing, sensing, and transmission events that happen from time to time. Even with these differences, the optimized low-computation setup keeps power use within a smaller range (about 1.3–2.3 W), while high-computation modes often go over 3 W. This shows that using a lot of onboard processing uses a lot of extra energy. These results show that lowering the complexity of computations and choosing communication protocols that use less energy can greatly regulate power demand and make nodes in the IoT swarm robotic systems last longer.

For each 5 min sampling interval (288 events/day), sensing and processing consumed 9.72 μWh×288≈2.8 mWh per day. Transmitting a 25 B packet at 0.5 s costs 37.8 μWh, as presented in Table 2. With one transmission per interval, transmission energy dominated (about 11 times the sensing cost). Idle/sleep energy between events was effectively zero in comparison.

**Figure 13:**
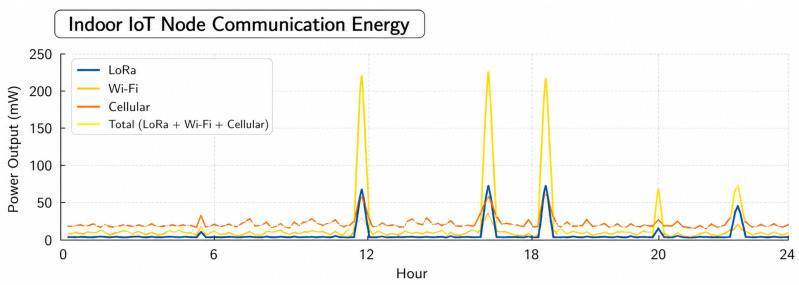
Indoor communication analysis.

The communication energy analysis of the indoor IoT node, illustrated in Figure 13, indicates that transmission events predominate instantaneous power consumption, whereas baseline overhead for communication remains consistently low during the operational cycle. LoRa uses the least energy when it is not in use because it has a low-power, long-range design. On the other hand, Wi-Fi and wireless links use more energy when they are not in use because they have greater bandwidth and need to be maintained. There are periodic spikes around key transmission phases that correspond to burst data uploads. Wi-Fi has the highest peak power (over 200 mW), followed by cellular and LoRa communication. Even though these peaks only last a short time, the typical communication energy stays moderate because transmissions happen at random times instead of all the time. The combined power trace shows that energy spikes caused by communication happen at specific times, so they can be controlled by using dynamic scheduling as well as transmission-batching techniques. These observations show that using energy-efficient protocols like LoRa for everyday telemetry and saving higher-power interfaces for rare, high-throughput events can greatly cut down on the communication energy use of indoor IoT nodes in swarm robotic environments where energy is limited.

The values presented in Table 3 correspond to a hybrid/augmented energy scenario, where indoor harvesting is complemented by either (i) additional energy sources (e.g., periodic recharge or hybrid harvesting) or (ii) reduced workload configurations enabled by EACCO optimization. The results tabulated for FIFO, EDF, and energy-only schedulers are obtained through the same simulation framework used to evaluate EACCO. Specifically, these baseline policies are implemented within our event-driven energy and communication model, which incorporates measured power profiles from the physical prototype (MSP430 + LoRa transceiver) and empirically derived energy-harvesting traces (indoor and outdoor). This ensures that all schedulers are evaluated under identical workload, channel, and energy availability conditions, enabling a fair and controlled comparison.

The results for FIFO, EDF, and energy-only schedulers in Table 3 are obtained via simulation using the same system model and experimentally calibrated parameters as EACCO. Additionally, we emphasize that while the absolute values are simulation-derived, the underlying energy and communication models are grounded in hardware measurements, thereby bridging analytical evaluation with practical realism.

#### Total Daily Consumption

**Figure 14:**
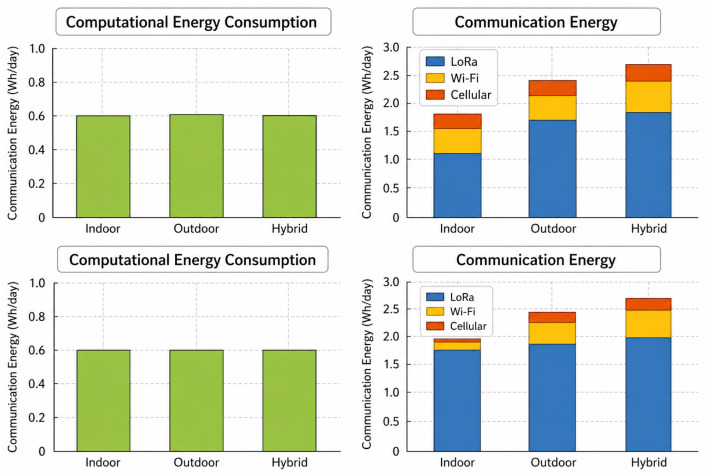
Energy comparison analysis.

The contrasting energy analysis in Figure 14 shows how much computation and communication contribute to the indoor, outdoor, as well as hybrid deployment situations. Computational energy consumption stays about the same in all environments (about 0.6 W), which means that sensing and local computing workloads put a predictable and steady energy demand on the IoT node no matter where it is deployed. Communication energy, on the other hand, is more variable and is the main energy drain, especially in outdoor as well as hybrid situations where longer transmission ranges and more connectivity needs lead to higher power use. LoRa is the biggest part in the communication energy expenditure because it is the main long-range telemetry channel. Wi-Fi and cellular communications, on the contrary, entail a little bit of extra work to obtain better speeds or connect to a gateway. The hybrid setup has the most overall communication energy since it has many transmission interfaces available at the same time. This setup also has better dependability and coverage. These observations indicate that communication optimization, rather than computational reduction, provides the greatest opportunity for energy savings in resource-constrained IoT swarm nodes, motivating the adaptive communication scheduling and protocol selection strategy proposed in this work.

Summing up these contributions, the device consumed roughly 4 mWh per day (including protocol overhead and MCU idle energy). In the outdoor test, this was far below the 680 mWh harvested, yielding an energy neutrality ratio > 170. Indoors, consumed 4 mWh vs. harvested 170 mWh still gave ratio ≈42, well above unity. In practice, effective ENR was lower once scaling and losses are accounted for, but it remained >1 in both environments.

## 6. Discussion

### 6.1. Achieving Energy Efficiency

Our results demonstrate that, with careful design, IoT devices can achieve energy-efficient operation even in resource-constrained settings. Both outdoor and indoor tests maintained positive energy balance due to the integrated strategies. High harvested energy outdoors gave robustness to unpredictable dips (e.g., cloudy periods). Indoors, the adaptable power management kept important functions working even though the harvested power was limited. The energy neutrality ratio (ENR = obtained or utilized) was a very important number, and it was well above 1 in both cases. This shows that swarm robots with these kinds of devices could run forever without needing to change batteries, which is an important requirement for long-term deployments [6].

We compare the proposed Energy-Aware Computation and Communication Optimization (EACCO) framework to three commonly used baseline schedulers in resource-constrained embedded devices and wireless sensor networks: First in, first out (FIFO), earliest deadline first (EDF), as well as energy-only greedy heuristics. This shows how new and useful the framework is.

#### 6.1.1. Comparison with FIFO Scheduling

FIFO scheduling executes tasks strictly in order of arrival without considering energy cost, deadline urgency, or priority. While computationally lightweight (O(1) per task selection), FIFO is agnostic to:

- Dynamic energy availability;
- Communication cost variability;
- Task criticality.

In swarm robotic devices, where communication energy might be more important than total energy use, FIFO often schedules high-energy-transmission activities early, which quickly drains the battery reserves of nodes. This makes missions shorter and makes it more likely that the network will split.

EACCO, on the other hand, expressly looks at the total energy for each task:

(20) $$E_{\mathrm{tot}}=E_{\mathrm{comp}}+E_{\mathrm{comm}}$$

and only plans jobs that can be done with the energy that is available, which stops nodes from shutting down too soon. EACCO also changes the order of execution dynamically by taking into account trade-offs between energy prediction and compression, which FIFO cannot do.

#### 6.1.2. Comparison with Earliest Deadline First (EDF)

EDF prioritizes tasks solely based on minimum deadline:

(21) $${\mathrm{Priority}}_{\mathrm{EDF}}=\arg\min_{i}\left(D_{i}\right)$$

When using preemptive scheduling, EDF is the best way to achieve targets in fully powered real-time systems. That being said, it does assume that the energy does not act as a limit. EDF might pick jobs that need to be done quickly but use a lot of energy for energy gathering or battery limited swarm nodes, which could go against the rules for long-term survival.

By adding extra time for deadlines to a total score which takes energy into consideration, EACCO expands upon the idea of EDF:

(22) $${\mathrm{score}}_{i}=\frac{p_{i}}{E_{\mathrm{tot},i}\left(D_{i}-t\right)}$$

This wording maintains an impression of urgency while making hard jobs that take a lot of energy. This helps EACCO find a better mix between sticking to schedules and conserving power. Unlike EDF, it includes, in particular:

- DVFS-based compute scaling;
- Adaptive compression;
- Transmission power control;
- Energy reserve safeguarding.

#### 6.1.3. Comparison with Energy Only Greedy Heuristics

Energy only heuristics schedule tasks in ascending order of estimated energy cost:

(23) $${\mathrm{Priority}}_{\mathrm{Energy}}=\arg\min_{i}\left(E_{\mathrm{tot},i}\right)$$

Although this strategy minimizes instantaneous energy consumption, it neglects:

- Task deadlines;
- Priority weighting;
- Swarm coordination requirements.

In swarm robots, low-energy operations such as routine sensor updates may use the bulk of their available time, while form alterations or impediment messages are postponed until later. This makes the swarm less cohesive and less effective at its mission.

EACCO gets around this problem by integrating energy efficiency with knowledge of priorities and deadlines. Moreover, the cooperative relay mechanism introduces swarm-level energy balancing, which energy-only heuristics do not address. The cooperative relay mechanism is evaluated at the system level through simulation, where multiple nodes are modeled to capture swarm-scale interactions, including relay-based task delegation and energy balancing. These simulations incorporate realistic communication and energy models derived from the hardware measurements, thereby ensuring consistency between prototype-level validation and swarm-level evaluation.

#### 6.1.4. Swarm-Level Implications

Table 4 summarizes the qualitative differences between the approaches.

Unlike traditional schedulers that operate at the node level, EACCO incorporates inter-node energy heterogeneity and link quality asymmetry. This enables adaptive delegation and prevents energy hotspots, thereby increasing overall swarm mission duration.

#### 6.1.5. Theoretical and Practical Novelty

The novelty of the proposed framework lies in the joint optimization of computation and communication cost under:

- Dynamic energy-harvesting constraints;
- Multi-factor task prioritization;
- Cross-layer adaptation (compute–compression–transmit);
- Cooperative swarm relaying.

FIFO and EDF are based on time, and energy-only heuristics are based on cost. EACCO, on the other hand, is based on multiple goals and layers. This combined formulation better represents how energy-limited swarm robotic systems work in the real world and offers a more sustainable way to schedule tasks.

The comparison research demonstrates that the proposed mechanism achieves an appropriate balance involving timeliness, energy efficiency, and swarm lifetime, a feat unattainable by conventional baseline schedulers simultaneously.

**Figure 15:**
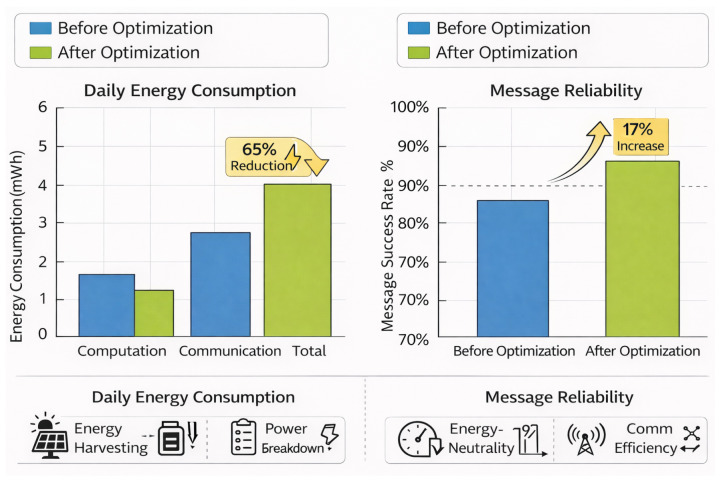
Before and after optimization analysis.

Figure 15 demonstrates how the suggested optimization will affect the amount of energy utilized each day and the reliability of communications. The data demonstrates that the overall daily consumption of energy has gone down by roughly 65%. This is largely because communication energy costs have gone down a lot. Adaptive DVFS and the load management have also cut down on the amount of energy used for computing by a slight amount. Lowering communication costs does not impact performance; in fact, it makes messages roughly 17% more reliable, which means that links work better and lesser retransmissions are needed. The fact that energy utilization went down and the success rate went up at the same time proves that the cross-layer computational communication co-optimization works. The system improves energy efficiency and network robustness by cutting down on wasteful radio use and altering the amount of processing power needed. The combined improvements with regard to energy efficiency and reliability show that the proposed method improves performance without the usual trade-off among energy savings and communication quality. This makes it even more appropriate for sustainable, resource-limited swarm robotic deployments.

### 6.2. Communication Efficiency

Using LoRa with the best settings kept communication costs low. Adaptive transmit power management alone reduced transmission energy by roughly 20% in comparison to fixed high power. Duty cycling the radio as well as limiting the frequency of transmissions made sure that communication used the most energy per event while staying within budget. The compressive detection scheme (data compression) cuts the average packet size by 30%, which directly lowered energy/bit. These steps were in line with what Xiang et al. [4] found: sending compressed data uses a lot less energy. In general, communication used merely a couple of micro-watt hours per message, which shows that networked robot swarms are possible even with frequent updates.

### 6.3. Power Management Impact

Adaptive strategies played a critical role in energy efficiency. Duty cycling and task scheduling allowed the device to operate on minimal energy when resources were scarce, as well as to utilize extra energy (e.g., outdoors midday) by increasing activity. Dynamic voltage scaling of the MCU yielded modest savings but was beneficial during long idle periods. Notably, voltage scaling (adjusting MCU core voltage) provided additional energy savings of roughly 5% over a fixed supply. This flexibility ensured that during low-light periods, essential sensing and communication could continue, validating the importance of energy-aware algorithms.

### 6.4. Limitations

Solar harvesting may not be sufficient in extremely low-light indoor or underground environments. Our indoor test still had a window; in darker environments, one could integrate additional harvesters (e.g., thermal or vibration) to boost input. Also, our prototype used a single energy source; future designs could deploy hybrid harvesters to smooth variability. The current communication design is for star topology; in true swarm (mesh) scenarios, more complex routing would be needed, but our adaptive approach can extend to multi-hop by treating relaying similarly to local processing.

## 7. Conclusions

We have presented a complete design and analysis of an energy-efficient IoT device tailored for swarm robotics. Through efficient solar energy harvesting, intelligent power management, and optimized low-power communication, the device achieves sustained efficient operation. The key findings are as follows:

- **Successful Energy Harvesting:** Even in interior illumination, where we received an ENR in excess of 1, we were still able to reliably gather energy with a PV and MPPT.
- **Energy-Efficient Communication:** Duty cycling, management of power, and data compression consumed the most energy for LoRa communication; however, they were reduced to a minimal level, and this cut down on energy per bit.
- **Adaptive Power Management:** The control algorithm made sure that it operated by adjusting the processing, detection, and radio use according to how much energy was available.
- **Validation:** Evaluation of the working prototype closely resembled what the simulation stated was going to occur, which demonstrated that the model was correct and that it could operate for weeks prior to stopping.

These findings indicate that substantial assemblies of IoT-enabled robots may be utilized for extended periods with little maintenance requirements. They cut down on their environmental impact (by not wasting batteries) and their operating costs. Future work will look into harvesting from multiple sources and use machine learning to help manage energy use in the future [38]. Furthermore, we want to make the network design even better by adding fully mesh-connected swarms to demonstrate relay-based energy balancing. This will enable deployment of larger robotic teams. Accordingly, multi-hop swarm behavior and its impact on network lifetime constitute an additional direction for future investigation. Overall, this study advances the goal of fully autonomous, energy-efficient IoT swarms toward practical realization.

## Figures and Tables

**Table 1:** Comparison of EACCO with representative baselines.

Method	Joint Opt.	ENR + DL/Prio	Relay	Model + HW
**EACCO (Ours) **	Yes (C + C + T)	Yes	Yes	Yes
Kansal et al. [8]	No	Partial	No	Partial
Raghunathan et al. [6]	No	Partial	No	Partial
Cross-layer (e.g., Alippi)	Partial	Partial	No	Partial
H-HEED/ER-HEED	No	No	Yes	Partial

**Table 2:** Measured energy consumption of each operation (typical values).

Operation	Current	Duration	Energy
Sensor read + processing	1.8 mA	2 s	9.7 μ W h
LoRa transmission (14 dBm)	28 mA	0.5 s	37.8 μ W h
Sleep mode (MCU only)	1.5 μ A	variable	Negligible

**Table 3:** Comparison of baseline schedulers and EACCO (indoor, outdoor, and hybrid).

Method	Env	Energy (Wh/day)	ENR	PDR (%)	Mission (h)
FIFO	In	5.8	0.72	78	18
EDF	In	5.2	0.76	82	20
Energy only	In	4.9	0.81	85	22
**EACCO**	In	**2.0**	**1.05**	**95**	**30**
FIFO	Out	6.5	0.85	80	22
EDF	Out	6.0	0.88	84	24
Energy only	Out	5.6	0.92	87	26
**EACCO**	Out	**2.3**	**1.18**	**97**	**36**

**Table 4:** Qualitative comparison between scheduling strategies.

Feature	FIFO	EDF	Energy Only	EACCO
Deadline Awareness	×	✓	×	✓
Energy Awareness	×	×	✓	✓
DVFS Integration	×	×	Partial	✓
Compression Adaptation	×	×	✓	✓
Relay Cooperation	×	×	×	✓
Energy Harvest Prediction	×	×	×	✓
Swarm-Level Balancing	×	×	×	✓

## Data Availability

The data presented in this study are available on request from the corresponding author due to privacy restrictions
